# Fecal microbiota transplantation results in bacterial strain displacement in patients with inflammatory bowel diseases

**DOI:** 10.1002/2211-5463.12744

**Published:** 2019-12-13

**Authors:** Manli Zou, Zhuye Jie, Bota Cui, Honggang Wang, Qiang Feng, Yuanqiang Zou, Xiuqing Zhang, Huanming Yang, Jian Wang, Faming Zhang, Huijue Jia

**Affiliations:** ^1^ BGI Education Center University of Chinese Academy of Sciences Shenzhen China; ^2^ BGI‐Shenzhen China; ^3^ China National Genebank Shenzhen China; ^4^ Shenzhen Key Laboratory of Human Commensal Microorganisms and Health Research BGI‐Shenzhen China; ^5^ Medical Center for Digestive Disease the Second Affiliated Hospital of Nanjing Medical University China; ^6^ Shenzhen Engineering Laboratory of Detection and Intervention of Human Intestinal Microbiome Shenzhen China; ^7^ Department of Biology Laboratory of Genomics and Molecular Biomedicine University of Copenhagen Denmark; ^8^ James D. Watson Institute of Genome Sciences Hangzhou China; ^9^ Key Lab of Holistic Integrative Enterology Nanjing Medical University China; ^10^ Macau University of Science and Technology China; ^11^ Present address: Department of Human Microbiome School of Stomatology Shandong China

**Keywords:** fecal microbiota transplantation, inflammatory bowel disease, random forest, shotgun metagenomic sequencing, strain displacement, strain‐level identification

## Abstract

Fecal microbiota transplantation (FMT), which is thought to have the potential to correct dysbiosis of gut microbiota, has been used to treat inflammatory bowel disease (IBD) for almost a decade. Here, we report an interventional prospective cohort study performed to elucidate the extent of and processes underlying microbiota engraftment in IBD patients after FMT treatment. The cohort included two categories of patients: (a) patients with moderate to severe Crohn’s disease (CD) (Harvey–Bradshaw Index ≥ 7, *n* = 11) and (b) patients with ulcerative colitis (UC) (Montreal classification S2 and S3, *n* = 4). All patients were treated with a single FMT (via mid‐gut, from healthy donors), and follow‐up visits were performed at baseline, 3 days, 1 week, and 1 month after FMT (missing time points included). At each follow‐up time point, fecal samples and clinical metadata were collected. For comparative analysis, 10 fecal samples from 10 healthy donors were included to represent the diversity level of normal gut microbiota. Additionally, the metagenomic data of 25 fecal samples from five individuals with metabolic syndrome who underwent autologous FMT treatment were downloaded from a previous published paper to represent fluctuations in microbiota induced during FMT. All fecal samples underwent shotgun metagenomic sequencing. We found that 3 days after FMT, 11 out of 15 recipients were in remission (three out of four UC recipients; 8 out of 11 CD recipients). Generally, bacterial colonization was observed to be lower in CD recipients than in UC recipients at both species and strain levels. Furthermore, across species, different strains displayed disease‐specific displacement advantages under two‐disease status. Finally, most post‐FMT species (> 80%) could be properly predicted (area under the curve > 85%) using a random forest classification model, with the gut microbiota composition and clinical parameters of pre‐FMT recipients acting as factors that contribute to prediction accuracy.

## Graphical abstract

Here, we profiled gut metagenomes of patients with inflammatory bowel disease before and after fecal microbiota transplantation (FMT) at both species‐ and strain‐level resolution. We investigated the principles of species engraftment 3 days after FMT using a randomForest model and identified the top contributable predictors to the accuracy of the model.

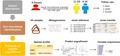

**Abbreviations**
AUC: area under the curve
CD: Crohn’s disease
FCM: flow cytometry
FMT: fecal microbiota transplantation
HBI: Harvey–Bradshaw Index
IBD: inflammatory bowel disease
rfcv: random forest cross‐validation
UC: ulcerative colitis

Inflammatory bowel disease (IBD) is a chronic inflammatory disease characterized by chronic immune‐mediated intestinal inflammation and consists mainly of Crohn’s disease (CD) and ulcerative colitis (UC). The etiology of IBD has been proposed to be multifactorial, involving a dysregulated immune response to environmental factors in a genetically susceptible individual 1. Interestingly, given the evidence accumulated in recent years, the gut microbiota is now recognized for playing an important role in IBD. Dysbiosis is a decrease in gut microbial diversity owing to a shift in the balance between commensal and potentially pathogenic microorganisms of the gut microbial ecosystem and has long been characterized as a trait of IBD patients 2, 3. The article by Sunkara *et al*. explains in detail about how gut microbiota dysbiosis is characterized by a significant reduction of obligate anaerobes and a sharp increase in facultative anaerobes. Release of anti‐inflammatory compounds is caused by a decrease in obligate anaerobes which causes increased inflammation 4. *Bacteroids fragilis* and *Faecalibracterium prausnitzii* were considered to have the potential to promote intestinal inflammation through downregulation of Treg cells 5, 6, 7.

Fecal microbiota transplantation (FMT) aims to modify the intestinal microbiota composition and function of the recipients by transferring donor fecal suspension into the gastrointestinal tract of a recipient and has become a promising method for manipulating the gut microbiota. Its successful application for the treatment of *Clostridium difficile* infection has inspired people to apply it to IBD patients 8, 9, 10, 11, 12, 13. However, this application is still in its early stages. According to a recent systematic review and meta‐analysis, after minimizing publication bias, IBD patients who received FMT had a remission rate of only 36.2%: 22% for UC and 60.5% for CD 14. Moreover, there is a lack of research regarding the efficiency and principles of FMT in treating IBD.

Clinical research to date has focused more on UC 11, 12, 13, and there has been insufficient research on the effects of FMT on CD patients, with only a few case reports and small‐scale case series reported 15, 16, 17, 18. In addition, the majority of studies conducted so far to investigate the role FMT plays in treating IBD have used 16S rRNA sequencing, which has limited resolution on taxonomic and functional classification of sequences. Contradictory results were often observed at species‐level resolution, making it hard to determine the exact role of different bacterial agents. For instance, the abundance of *F. prausnitzii* was found to decrease in one study and to increase in another 19, 20. Thus, it is necessary to be able to appreciate the whole composition of gut microbiota at a strain level. Strain‐level variants within microbial species are crucial in determining their functional capacities within the human microbiome, such as interaction with host tissues 21, modulation of immune homeostasis 22, and xenobiotic metabolism 23. Shotgun metagenomic sequencing with the ability to target all DNA material in a sample can give a base pair‐level resolution of the genome that makes single nucleotide analysis possible. Additionally, promising machine learning methods could enable the establishment of predictive models to predict the microbiota composition of post‐FMT recipients. Recently, Smillie *et al*. constructed a machine learning model to predict the species profile of post‐FMT recipients for 18 *C. difficile* patients and found that bacterial abundance and phylogeny were the strongest determinants of engraftment 24. In our study, we utilize a random forest model to predict the mOTU profile of IBD recipient 3 days after FMT and identified the variables that contribute most to model prediction accuracy.

## Materials and methods

### Patient recruitment and donor selection

Patients aged 19–64 years with moderate to severe CD, as defined by Harvey–Bradshaw Index (HBI) and UC, as defined by Montreal classification, were recruited from the Second Affiliated Hospital of Nanjing Medical University, China, from 2012 to 2014. Exclusion criteria included: (a) patients accompanied with serious diseases, including other intestinal diseases; (b) patients with refractory obstruction symptoms after conservative treatment; and (c) patients who received biological therapies had uncertain clinical response 3 months before FMT. Clinical metadata of IBD patients—including anthropometric index, clinical parameters, and blood test results—were obtained at each follow‐up time point.

Donors were either related (genetically related family members) or unrelated (screened unrelated family members). Donors did not use antibiotics, laxative, or diet pills in the past 3 months and had no recent gastrointestinal diseases. Donors with any history of illness especially those diseases or conditions potentially associated with specific changes in gut microbiota were excluded. All the donors were assessed by laboratory evaluation and biochemical test. Besides, donor’s family health history, personal psychological health, and living environment were assessed. Detailed standards of patient recruitment and donor screening were previously published 17.

### Stool sample collection and FMT procedure

**Figure 1:**
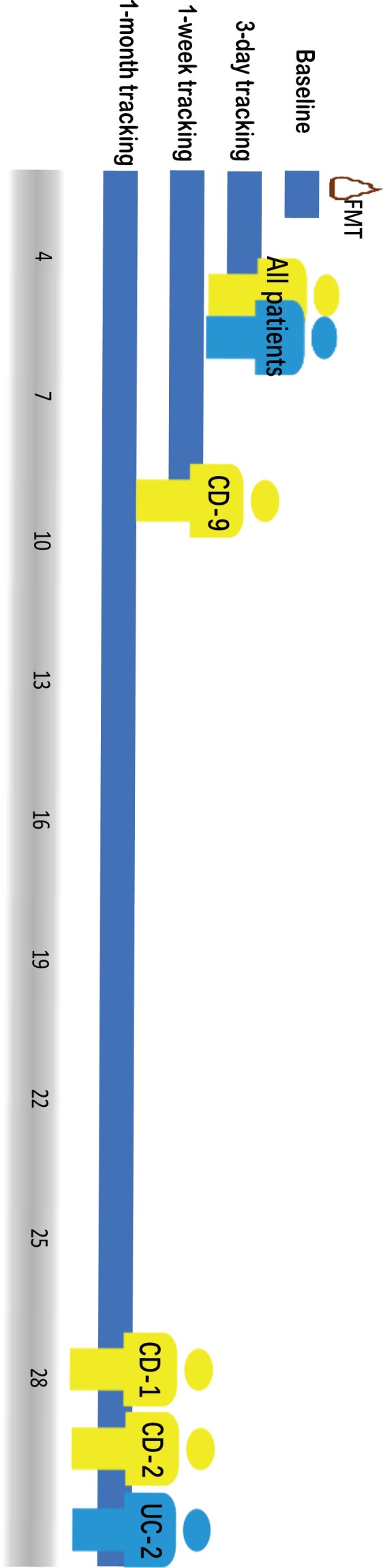
Study design and follow‐up visits of the patients. Patients were labeled with disease subtype CD‐ or UC‐ as a prefix plus a random assigned number as suffix.

The dataset was composed of 10 fecal samples from 10 healthy donors, among which six were FMT donors, and 34 fecal samples from 15 IBD patients. Donor fecal samples were collected prior to FMT in the same batch, and fecal samples from the same healthy donor were collected at the same time point. Stool samples from recipients were collected at baseline, day 3, and day 7 (or day 30) (Fig. 1). For autologous FMT treatment, 25 additional fecal samples from five metabolic syndrome individuals were obtained from the Vrieze *et al*. 25 study with follow‐up points on day 0 and days 2, 14, 42, and 84 after FMT. In summary, 34 samples were used for the analysis of the allogenic FMT group, 25 for the autologous, and 10 for the healthy group.

Fecal samples were obtained from scanned donors and were isolated for microbiota at laboratory. Fecal microbiota from the donor was prepared according to the manual method of filtration, centrifugation, washing, discarding, and resuspension and repeated processes. Purified fresh fecal microbiota suspension was input into patients’ mid‐gut by a tube within gastroscope under anesthesia, and the entire procedure should be done within 1 h.

### Blood test for lymphocyte population

Blood and stool samples were collected at the same time and were analyzed by flow cytometry (FCM) and laboratory examination, and clinical activity was also assessed at each visit. Inflammation markers we used are C‐reactive protein, CD3^+^, CD19^+^, (CD3^+^, CD4^+^), (CD3^+^, CD8^+^), and (CD16^+^56^+^). Details were previously published 17.

### Metagenomic sequencing and processing methods

DNA extraction and metagenomic sequencing of IBD fecal samples and healthy fecal samples were performed at BGI‐Shenzhen, China, following HiSeq 2000 sequencing protocol. Metagenomic sequencing of autologous FMT treatment samples was performed at the Genomics Core Facility of the European Molecular Biology Laboratory, Heidelberg, Germany using HiSeq 2000.

Illumina sequencing reads were quality controlled by trimming low‐quality bases (quality score < 20), filtering adapter reads, and removing host‐related reads after mapping to the human genome database. The reads quality control procedure was conducted using cOMG with default parameters 26. After quality control, 1 379 430 125 sequences were obtained, with a mean of 31 350 685 sequences per sample.

### Microbiota taxonomic profiling

Species‐level quantification of metagenomic sequencing reads was achieved using mOTU software with default parameters. mOTUs is a method that establishes metagenomic operational taxonomic units based on single‐copy phylogenetic marker genes. It maps the quality‐controlled metagenomic sequencing reads against the m‐OTUS.v1.padded database, which is composed of 10 MGs extracted from 3496 prokaryotic reference genomes (download from NCBI) and 263 publicly available metagenomes (from the MetaHIT and HMP projects), and then outputs metagenomic OUT linkage groups (m‐OTUS) 27.

For strain‐level profiling, metaSNV was utilized to process quality‐controlled metagenomic sequencing reads. metaSNV is a method that is able to disentangle conspecific strains in metagenomic samples using specific single‐site allelic variation (SNVs). It uses a collection of microbial reference genomes in which each species is represented by a single representative genome or gene collection 28. To maintain consistency with previous species profiles, we specified the m‐OTUS.v1.padded database as our reference genome or gene collection during this procedure. First, we mapped quality‐controlled sequencing reads to the m‐OTUS.v1.padded database using bwa and Ngless. Next, we ran qaCompute on each sample to determine the average coverage over each reference in each sample and aggregated the coverage information. We then took advantage of the mpileup tool to compute genomic variation and outputted all the variant positions that met the default‐imposed quality criteria. Lastly, we computed per species pairwise distance matrices for the samples.

### Quantification and Statistical analysis

All statistical analyses were performed in R using the following packages: vegan, Hmcc, pROC, and randomForest. We conservatively used only the baseline and day 3 time point samples for each patient when conducting all the two‐sided statistical tests.

#### Diversity comparisons

The diversity of each gut microbiota community per sample was calculated based on its mOTU profile, referred to as the Shannon index, using the vegan package. The Kruskal–Wallis test was used as a significance test for this multigroup comparison.

#### Species‐level changes after FMT

After species profiling all fecal samples using mOTU, we took only the species with a detected relative abundance of at least 0.001 into account to avoid ambiguous results. In order to determine whether donor microbiota could be transferred to recipients, we divided the microbiota composition of post‐FMT recipient into four groups: donor‐specific species, recipient‐specific species, common species (shared by donor and recipient), and new species (not found in either the donor or the pre‐FMT recipient). We quantified these four groups by comparing the gut microbiota mOTU profiles of the pre‐FMT recipient, the post‐FMT recipient, and the donor. Results were visualized using bar plot with all available follow‐up time points.

#### Community‐level changes after FMT

Community‐level changes in gut microbiota composition between pre‐FMT and post‐FMT recipients were represented by the Bray–Curtis distance, which was computed using the vegan package after applying a logarithmic transformation to mOTU relative abundance with the function log(*x* + *x*_0_), where *x* is the original relative abundance of a certain mOTU and *x*_0_ = 1e‐6. The cosine dissimilarity was also used to examine the correlations between gut microbiota compositions pre‐FMT and post‐FMT, and between post‐FMT recipients and donors. Results were displayed using scatter plots.

#### Strain‐level changes after FMT

Strain differentiation, which was determined by comparing the presence or absence of donor‐specific, recipient‐specific, and previously undetected SNVs, was monitored in post‐FMT recipients based on the output files of metaSNV. Similar to the process of determining species retention and transplantation, the gut microbiota composition of post‐FMT recipients was categorized into three groups: donor‐specific strains, recipient‐specific strains, and common strains (shared by donor and recipient). We excluded the newly gained strains because that was not of interest here. Quantification of the three groups was determined according to the frequency per filtered SNVs set.

#### Species engraftment model

We sought to investigate whether the microbiota composition of post‐FMT recipients could be predicted using advanced machine learning models. We therefore applied the random forest algorithm in R to predict the presence (random forest classification model) and abundance (random forest regression model) of each mOTU in every post‐FMT recipient sample. For a dataset comprised of 15 samples and 123 filtered mOTUs, these models are trained on 15 × 127 total instances. The inputs for these predictions are the gut microbiota composition of each pre‐FMT patient and their corresponding donor at a species level, along with clinical metadata of the pre‐FMT recipient and donor. Random forest is a collection or ensemble of classification and regression trees trained on targeted datasets. It is resistant to overfitting and is considered stable in the presence of outliers. The error rate of the classification of all the test sets is the out‐of‐bag estimate of the generalization error 29.

First, we eliminated the condition of class imbalances by filtering out mOTUs that existed in less than three samples to avoid prediction bias in favor of the majority class. Second, the mtry parameter with the lowest error was picked using the random forest cross‐validation (rfcv) function with fivefold cross‐validation to avoid overfitting problem. Third, we applied the randomForest function to perform classification of post‐FMT recipients across all mOTUs. This resulted in 123 randomForest classification models in total, and we computed the area under the curve (AUC) value for each model. Finally, we chose important features from those models that had good prediction performance (AUC bigger than 0.9).

For the regression model, we also accounted for class balance and then used the rfcv function with the same predictors that we used in the classification model to perform prediction.

#### Feature importance

To minimize the bias caused by different value scale induced by adding phenotype information as predictors, random forest calculates feature importance by removing each feature from the model and measuring the decrease in accuracy (for presence) or the increase in the mean‐square error (for abundance). According to these importance scores, we ranked features in decreasing order across models and picked 40 with the highest scores to display.

#### Correlations between change in mOTUs and in clinical parameters

Clinical metadata of patients was collected at baseline and follow‐up visits, including physical parameters, inflammation markers, lymphocyte population, blood fat, and immunoglobulin. Blood lymphocytes were analyzed by FCM at later phase, and patients were excluded from the analysis for having been treated with immunomodulators or steroid when presented to our hospital. To avoid the possible bias which might be caused by the non‐normality nature of data pairs, we used the rcorr function in the Hmisc package to compute the Spearman rank‐order correlation instead of Pearson correlation iterating from each mOTU–clinical index pair. The change in each mOTU was defined as the increase or decrease in its relative abundance 3 days after FMT treatment compared to baseline. Changes in clinical index were computed based on the absolute score recipients got at baseline and 3 days after FMT treatment. For multiple comparisons, the Benjamini–Hochberg method was used to adjust the *P* value to control for false positives. Lastly, we drew a network using Cytoscape based on the pairs with a *q*‐value smaller than 0.05 30.

### Ethical statement

This study was carried out in accordance with the recommendations of good clinical research practice, the Ethical Committee of the Second Affiliated Hospital of Nanjing Medical University, and BGI‐IRB (BGI‐R004‐05). The protocol was approved by the Ethical Committee of the Second Affiliated Hospital of Nanjing Medical University and BGI‐IRB. All subjects gave written informed consent in accordance with the Declaration of Helsinki.

## Results

### Bacteria characterization at a species level

**Figure 2:**
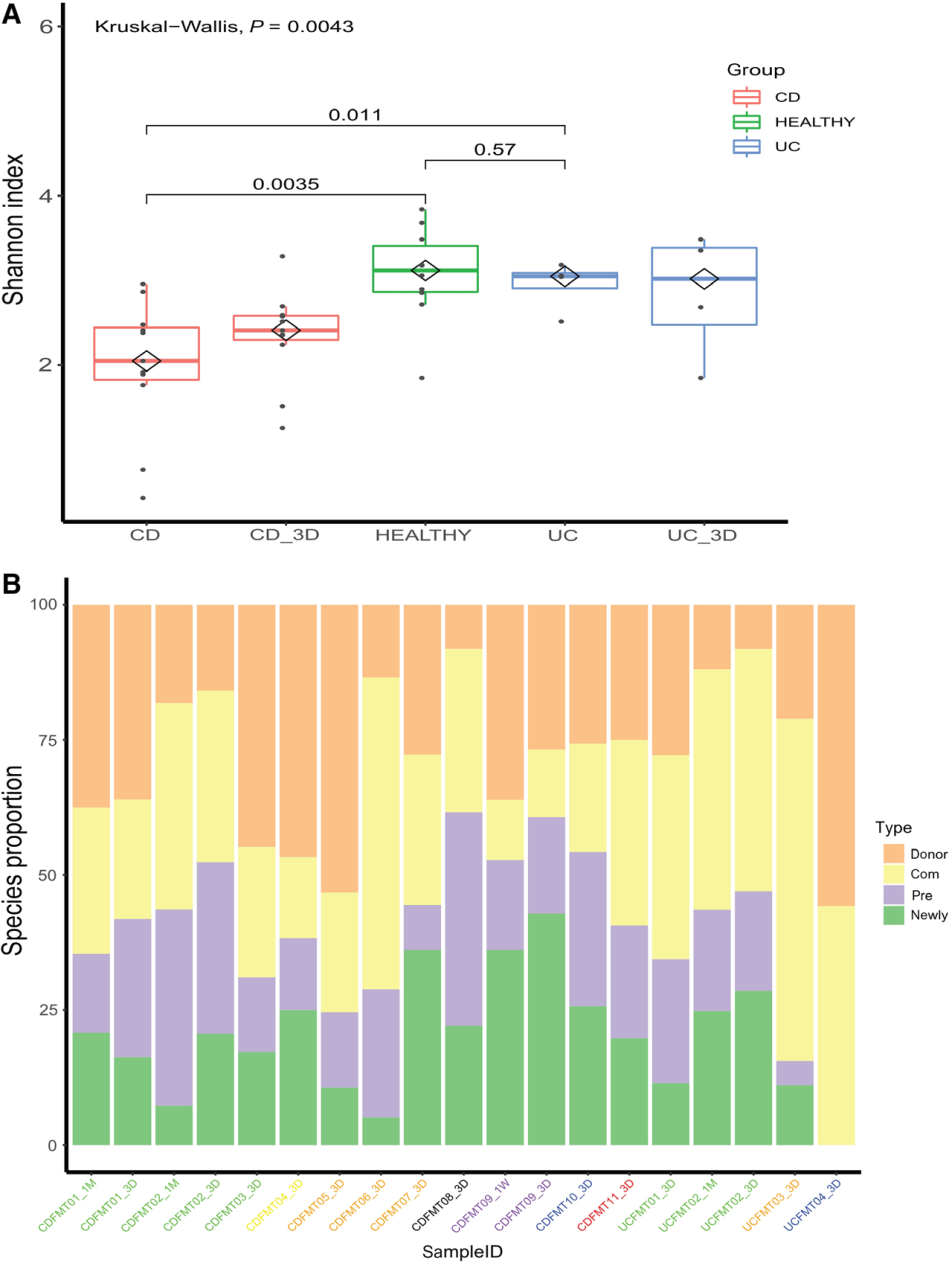
Bacterial communities undergo compositional changes in IBD recipients after FMT. (A) The Shannon index of gut microbiota was lower in IBD patients than in healthy controls, and was not significantly improved 3 days after FMT (*P*‐value > 0.01). Different groups are represented by different colored boxes. (B) The proportion of species gained from the donor in post‐FMT recipients lasts during follow‐up visits. However, the proportions varied among recipients, even those who shared a donor (labels with the same color). Gut microbiota composition per patient was divided into four parts: orange represented donor‐specific species, yellow represented species shared by donor and recipient, purple represented recipient‐specific species, and green represented newly gained species.

After profiling sequenced fecal samples using shotgun metagenomics, the Shannon index (alpha diversity of a community) of gut microbiota was measured across IBD recipients. Results showed that the average Shannon index of CD patients was significantly lower than that of healthy controls (*P*‐value = 0.0035). In UC patients, although their Shannon index was lower than the average in healthy controls, dysbiosis was not significant (*P*‐value = 0.57). Although the result was consistent with previous study 13, it should also be contextualized from the small sample size of UC cohort. Three days after FMT treatment, the average Shannon indexes of both CD and UC recipients had not significantly improved (*P*‐value > 0.01) (Fig. 2A). Unexpectedly, CD‐6, CD‐7, CD‐8, and UC‐2 had a decreased Shannon index.

Among the whole population of the gut microbiota, some bacteria may be more important than others for maintaining a healthy gut environment. For example, 3 days after FMT treatment, there was a universal increase in *Bacteroides* that have been shown to exist at lower levels in IBD patients than in healthy donors 31. Some highly individualistic performances were also observed: CD‐9 gained an abundant amount of *Lactobacillus*, which was considered to be probiotics, and CD‐1 had a great decrease in *Citrobacter*, which was recognized to be pathogenic bacteria (Fig. S2). The amounts of species each recipient gained from their donor after FMT are shown in Fig. S1.

### Bacterial engraftment at the species level

To investigate the extent to which the gut microbiota of recipients could be altered by FMT treatment, we evaluated both the degree and direction of change. Results showed that microbial communities underwent large compositional changes after FMT, and these changes persisted throughout follow‐up visits (Fig. 2B).

On average, post‐FMT CD recipients gained 29.4% of mOTUs from donors (*n* = 11, SD = 14.4%), while post‐FMT UC recipients gained 28.2% of mOTUs from donors (*n* = 4, SD = 20%). Our results were analogous to a previous study that found that FMT recipients gained 35% of mOTUs from donors (*n* = 436, SD = 27%) 28.

**Figure 3:**
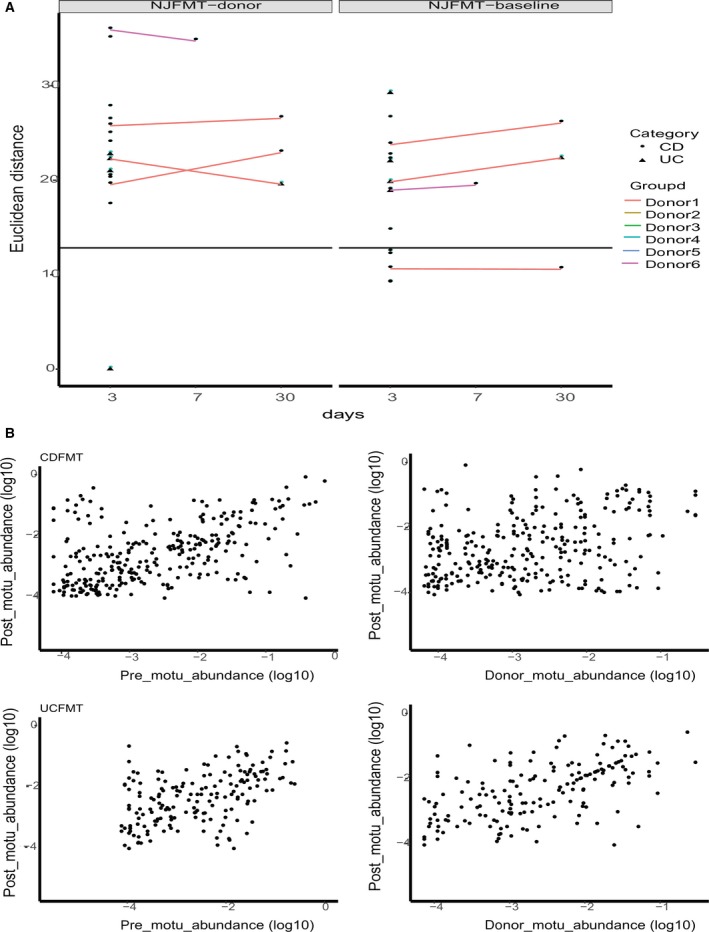
High compositional resemblance of the gut microbiomes of post‐FMT recipients and their prestatus, as well as post‐FMT recipients and their donors. (A) After FMT, the microbiota composition of most patients is further from their initial status than natural shift observed in placebo (solid black line). Additionally, recipients with the same donor (lines of the same color) may vary in their shifting tendency. (B) High consistency (median cosine similarity > 0.9) is found between post‐FMT IBD patients (3 days after treatment) with their pre‐FMT status, as well as with their donors.

By measuring the distance between donor–recipient pairs using Euclidean distance, we determined the direction of microbiota change. Results varied between different donor–recipient pairs. Out of the four patients that had two follow‐up time points, we found that CD‐9 and UC‐2 tended to be closer to their donors and further from their pre‐FMT status. CD‐2 showed a slight tendency to return to their initial status, but the disturbance was small enough to be ignored (a shift from 10.628–10.57). Surprisingly, CD‐1 showed an increased distance from both their donor and their pre‐FMT status, which could be attributed to environmental factors. Though CD‐1, CD‐2, and UC‐2 all shared the same donor, the direction of their gut flora shift after the treatment varied (Fig. 3A). In addition, we explored the abundance consistency of mOTUs of recipients before and after FMT. mOTUs of the recipient post‐FMT were highly correlated with mOTUs of the recipient pre‐FMT (median cosine similarity of UC patient mOTUs = 0.93, CD patients = 0.95). More importantly, the results showed that mOTUs of post‐FMT recipients had high similarity to mOTUs of their donors (median cosine similarity of UC patient mOTUs = 0.95, that of CD patients = 0.91) (Fig. 3B).

### Bacterial engraftment at the strain level

**Figure 4:**
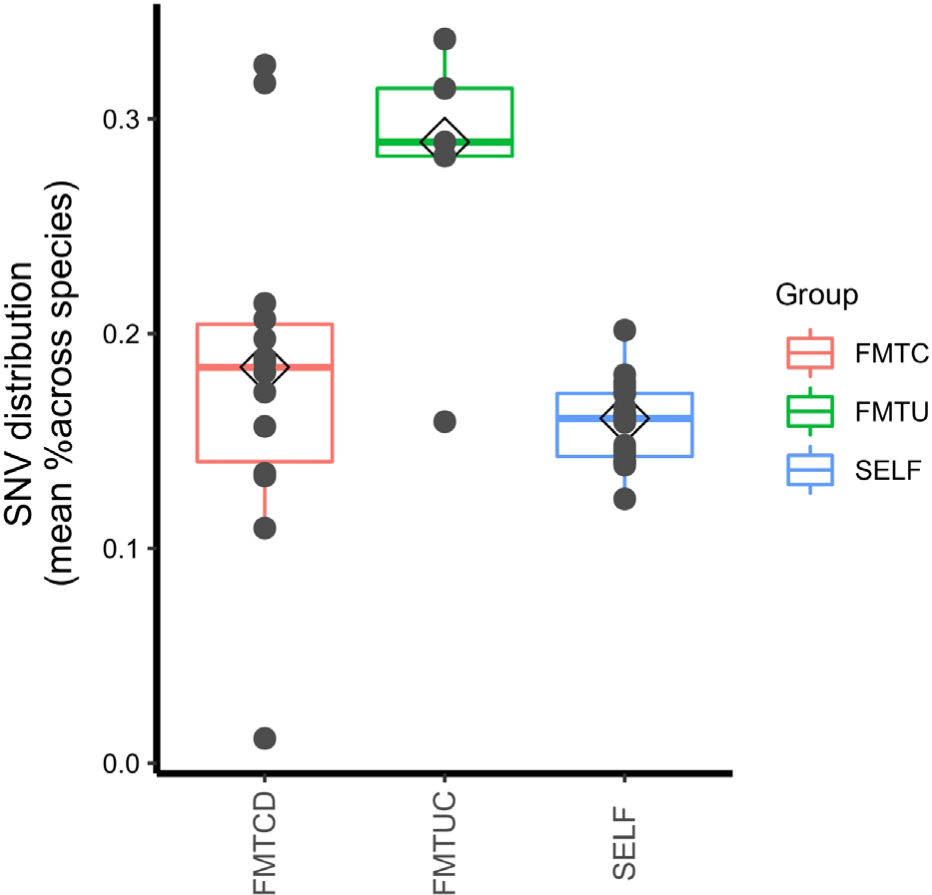
UC recipients display higher strain‐level variations than CD recipients 3 days after FMT treatment. SNVs of UC and CD recipients after FMT treatment are a bit higher than autologous FMT recipients (*P*‐value = 0.148 and 0.234, respectively). SNVs of UC recipients are significantly higher than CD recipients after FMT treatment (*P*‐value = 0.00056).

To investigate the extent of strain‐level changes in our study groups, we monitored SNVs identified at baseline over all available time points. Higher levels of SNVs were observed in UC FMT recipients and CD FMT recipients compared to autologous FMT recipients from a previous paper 25 (*P* = 0.0056 and 0.148, respectively). Moreover, SNVs were found to be higher in UC FMT recipients than in CD FMT recipients (*P* = 0.070; Fig. 4).

**Figure 5:**
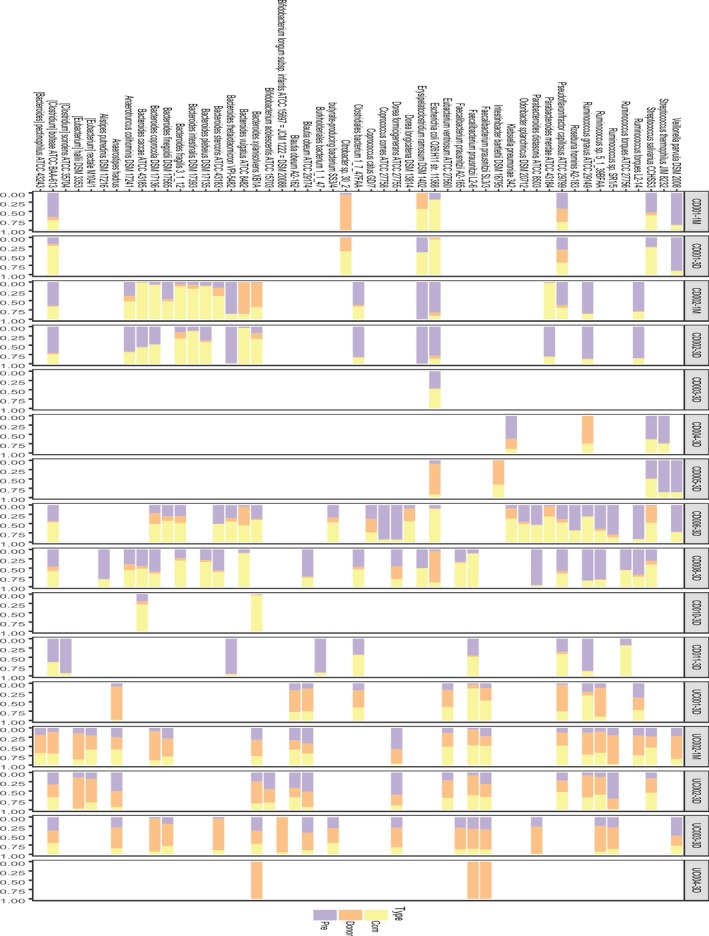
Some donor‐specific strains undergo transfer, and the existence of donor strains is highest 3 days after FMT. The rate of donor strain transfer is greatest in recipients 3 days after FMT (UC: 62.8 ± 25.3%, CD: 11.4 ± 10.3%), and a portion of them persists in recipients 1 month later (UC: 46.9%, CD: 19.99 ± 10.1%). Proportions of donor‐ and recipient‐specific strains across 50 species are shown in orange and purple, respectively.

To investigate whether this increased variation was due to the transfer and establishment of donor microbiota, we followed methods described in a previously published paper 32, defining a set of determinant genomic positions (containing both donor‐ and recipient‐specific SNVs) and monitoring them over time (Fig. 5). For the credibility of SNV detection, we chose species with sufficient abundance that were consistently detected in at least one donor–recipient pair. Donor‐specific SNVs were most highly retained 3 days after FMT (UC: 62.8 ± 25.3% of determinant positions across recipients, CD: 11.4 ± 10.3%) and were still present 1 month later (UC: 46.9%, CD: 19.99 ± 10.1%). This was in contrast with the much lower rates of variation observed at equivalent time points in autologous FMT recipients (9.5 ± 1.8%) (Fig. S1), showing that the increased variations of gut microbiota in post‐FMT patients could be attributed to donor strain transfer instead of temporal variability.

**Figure 6:**
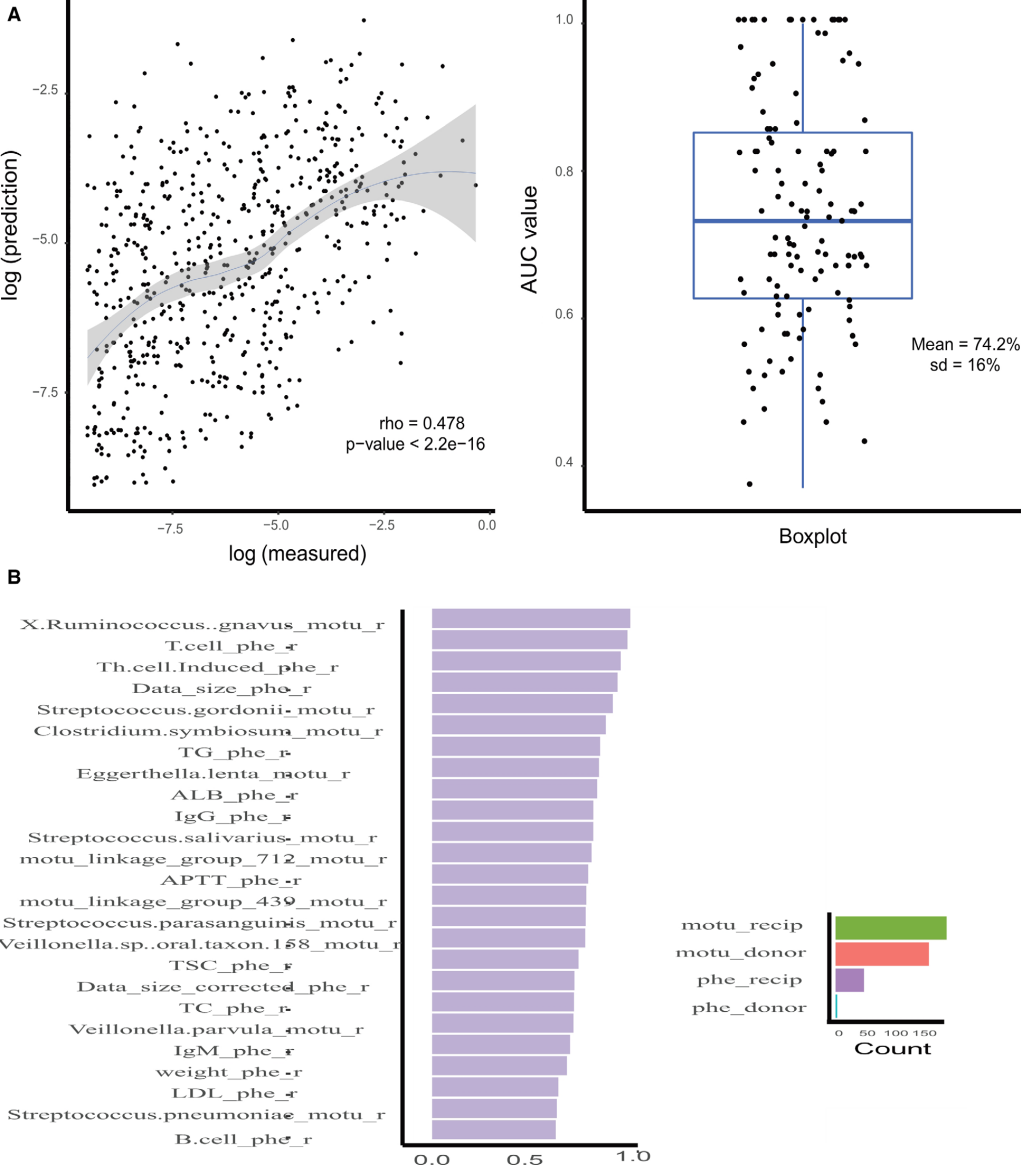
Random forest models have the ability to predict the gut microbiota composition of post‐FMT patients. (A) Left panel shows the classification result: Predicted values have a moderate consistency with true values (ρ = 0.478 and *P*‐value < 2.2e‐16). Right panel shows the regression result: a boxplot of all the AUC values of each mOTU in post‐FMT recipients (median AUC value = 74.2%, SD = 16%). (B) Important variables are computed across those models, defined as those with an AUC value greater than 0.90. Important variables are divided into different categories (represented by different colors). The top 25 variables are classified as the clinical parameters of recipients.

Furthermore, marked differences in colonization success were observed between UC and CD recipients who shared a donor (subjects CD‐1,2,3,8, and UC‐1,2). Three days after treatment, UC‐1,2 retained a higher amount of donor‐specific SNVs compared to CD‐1,2,3,8 (48.9%, 44.4%, 11.9%, 3.4%, 1.5%, and 9.3%, respectively). Extensive coexistence of donor and recipient strains (CD: in 44.1 ± 17.1% of shared species, UC: 21.3 ± 14.1%) was found in all other recipients and persisted for at least 1 month. This suggests that novel strains can colonize the gut without replacing the indigenous strain population of the recipient. It appeared that introduced strains were more likely to be established in a new environment if the species was already present, and a pattern of donor strains establishing alongside indigenous strains of the recipient was observed. While the phenomenon of donor strain establishment occurred in both CD and UC recipients, UC patients were more susceptible to external sources of microbiota (Fig. 6).

Donor strains showed different transferability under different disease status. Donor‐specific strains like *Ruminococcus torques* ATCC 27756,* Ordoribacter splanchinicus *DSM 20712*, Klebsiella pneumoniae *342*, Intestinaibacter bartlettii *DMS 16795*, Escherichia coli *O26:H11 str. 11368*,* and *Erysipelatoclostridium ramosum *DSM 1402 only exerted strain displacement in CD patients, while donor‐specific strains like *F. prausnitzii *SL3/3,* Eubacterium ventriosum *ATCC 27560,* Blautia obeum *A2‐162,* Bifidobacterium longum subsp.infantis *ATCC 15697 = JCM 1222 = DSM 20088,* Anaerostispes hadrus*, and *Eubacterium rectale *M104/1 only exerted strain displacement in UC patients (Fig. 5).

### Construction of a prediction model for gut microbiota composition of post‐FMT patients

According to what we have discovered in previous species‐level analysis, microbiota of post‐FMT recipients are a complex mixture of species from the donor, species from the recipient, and species gained from the environment. We speculated that after accounting for the gut microbiota composition of pre‐FMT recipients and donors, along with the corresponding clinical metadata of the recipients, we might be able to predict the post‐FMT gut microbiota of the recipients. We, therefore, performed random forest classification and regression analysis, which is nonlinear and can accept categorical and continuous predictors simultaneously from our data 29.

**Figure 7:**
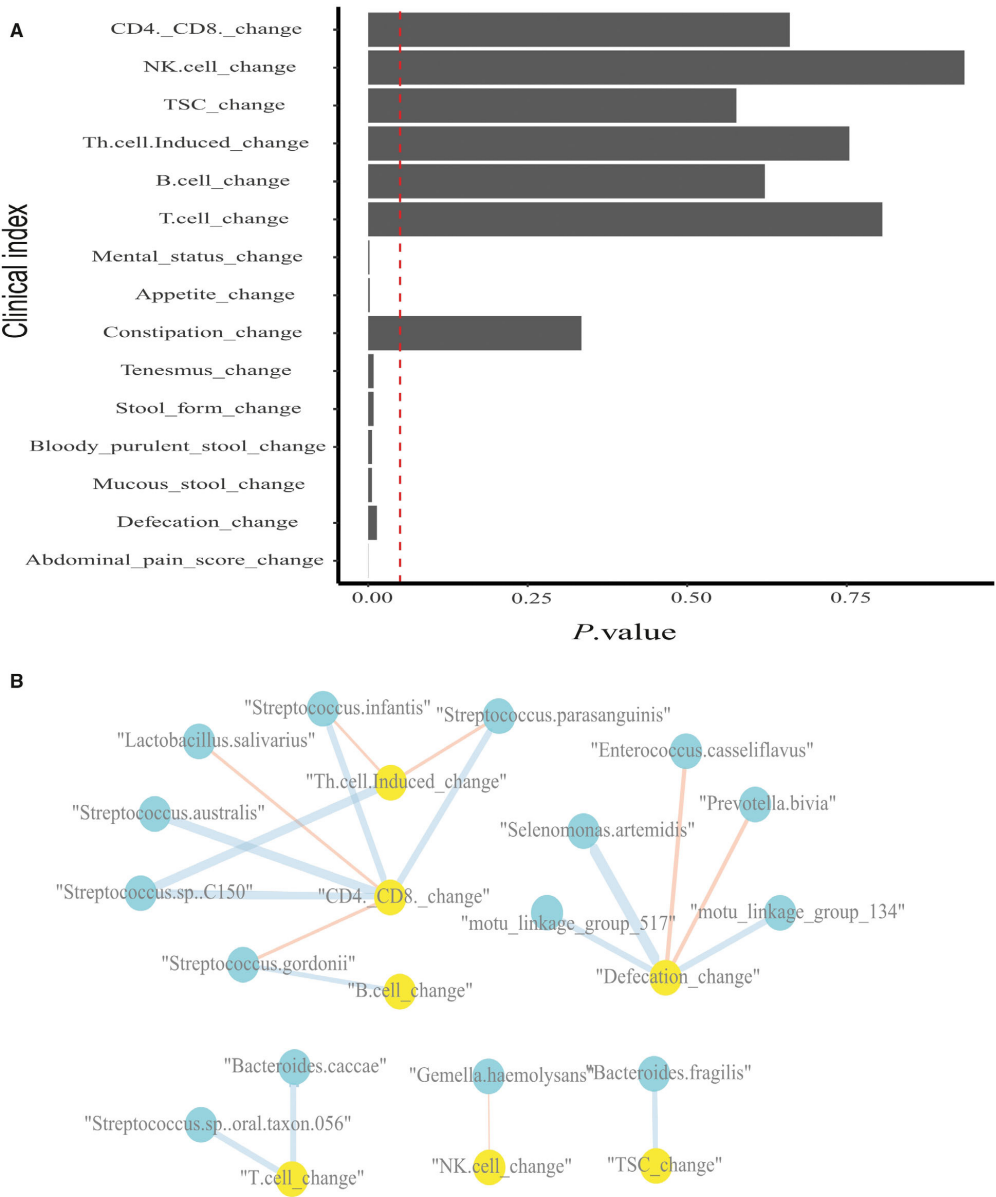
Some clinical indexes of IBD recipients have significantly changed 3 days after FMT, and several clinical indexes correlated with changes in the mOTU profiles of recipients. (A) Mental status, appetite, tenesmus, etc. significantly changed 3 days after FMT (*P*‐value < 0.05). Vertical dotted line indicates a *P* value of 0.05. (B) Defecation changes and CD4^+^/CD8^+^ changes have relationships with several mOTUs. Blue represents a significant positive correlation, while red indicates a significant negative correlation (*P*‐value < 0.01). Blue indicates gut microbiota species, while yellow indicates clinical indexes. The width of the lines indicates the weight of correlation.

To investigate whether species compositions of post‐FMT patients—that is, the mOTUs profiles—were predictable, we first examined the presence of each mOTU across post‐FMT recipients using the randomForest classification model and computed the average AUC (mean = 74.2%, SD = 16%). We then utilized a randomForest regression model to test the predictability of abundance of each mOTU (ρ = 0.478, *P* < 2.2e‐16). Results indicated that the presence of most (> 80%) species of post‐FMT recipients was highly predictable (AUC > 85%), while a small portion of species was not. The abundance of mOTUs of post‐FMT recipients was moderately predictable (Fig. 7A). Our results were poorer than a similar study conducted by Smillie *et al*. 24 on 19 recurrent *C. difficile* infection patients. One possible explanation for this discrepancy may be that they included other predictors in their model construction in addition to the ones we used: taxonomy, abundance, clinical metadata, sequencing depth, genome statistics, physiology, and resource utilization.

The randomForest model also provided an algorithm to rank the contribution of each predictor based on variable importance score. According to our analysis, among the top 40 most important variables (see Materials and methods), the IgA score, T‐cell, and Th‐cell‐induced of the recipients were the top three clinical‐related elements. *Streptococcus anginosus, Bacteroides plebeius, Clostridium bolteae, Streptococcus thermophilus*, and *X. Ruminococcus gnavus* were the top five species in the classification model (Fig. 7B). In terms of species‐related factors, *S. anginosus* was reported to be associated with colorectal cancer and *Ruminococcus gnavus* was found to be linked with a certain type of immunological rejection.

### Clinical outcomes

Out of all 15 patients, 8 out of 11 CD patients and three out of four UC patients were relieved 3 days after FMT treatment. Clinical improvement was defined as a decrease in the HBI > 3 for CD and a decrease in the Mayo score > 3 for UC (Table S1). And there were no severe or obvious adverse events during endoscopic infusion, after FMT, and the short‐term follow‐ups for those 15 IBD patients.

### Relationship between changes in clinical index and changes in gut microbiota

Potential antigens in the microflora could have pro‐ or anti‐inflammatory effects, and it could be argued that by reacting to these antigens, an organism is mounting an autoimmune response; by extension, the chronic mucosal inflammation of IBD could be thought of as an autoimmune disease. Given this perspective, it would make sense to relate the change in clinical parameters to the abundance change of gut microbiota in response to FMT treatment. We established the relationships between the change in clinical indexes and in mOTUs of recipients using Spearman’s correlation. We found that defecation changes were significantly positively correlated with *Selenomonas artemidis* and two unclassified species, and negatively correlated with *Enterococcus casseliflavus* and *Prevotella bivia*. Changes in CD4^+^/CD8^+^, which have been identified to be higher in IBD patients than in normal people in previous studies, were significantly positively correlated with *Streptococcus.*sp. *C150*, *Streptococcus infantis*, *Streptococcus parasanguinis*, and *Streptococcus australis*, and negatively correlated with changes in *Streptococcus gordonii* and *Lactobacillus salivarius*, a probiotic bacterium that lives in the gastrointestinal tract and has a range of therapeutic properties including suppression of pathogenic bacteria 33. Changes in TSC were significantly positively correlated with changes in *B. fragilis*, which is found in most anaerobic infections and can promote the induction of type 1 T helper (TH1) cells, suppress IL‐17 production, and improve experimental colitis 34. Additionally, we tested whether the physical characteristics of patients, such as BMI, age, and disease duration, (Table S2) could affect clinical outcomes. Changes in CD4^+^/CD8^+^, Th.cell.Induced (counted by FCM), and abdominal pain score were found to significantly negatively correlate with the disease start age of patients (*P* < 0.05), which could reflect disease duration. In addition, changes in CD4^+^/CD8^+^ and Th.cell.Induced were significantly negatively correlated with the age of patients (when patients received FMT treatment). Disease duration and the age of patients were also discovered to be important features in the random forest classification model. As a result, we speculated that disease duration and age could be used as stratifying factors for IBD patients in future therapy plans (Fig. 7B).

## Discussion

Consistent with previous findings, our study found reduced bacterial diversity in CD and UC patients. Strain‐level analysis monitored across samples revealed that 3 days after FMT treatment, a certain amount of species had noticeable strain replacements. Moreover, donor‐specific strains belonging to different species demonstrated differentially competitive advantages during the process of displacement, measured by their relative abundance in recipients after FMT. We also observed that same‐donor recipients undergo varying degrees of gut microbiome shifts, implying that the FMT treatment effect may be patient‐specific, and raising the possibility of patient stratification in clinical application.

We also aimed to identify factors that could contribute to the accurate prediction of post‐FMT gut microbiota composition of the recipients. The moderate predictability of the classification and regression model suggests that the gut microbiota composition of post‐FMT recipients can be recognized not through sequencing methods but through algorithms, indicating a promising future toward FMT precision treatment. In our model, we only take the species composition of the donor and pre‐FMT patient, along with clinical indexes of the pre‐FMT patient as predictors. There is space left to enhance the resolution of prediction accuracy. Based on previous studies concerning the etiology of IBD, factors like genetic background, nonbacterial components (virome, fungi), metabolites profile, and dietary records have the potential to account for the unexplainable part of our model.

Associations between immunological factors and clinical outcomes provide us with some limited but intriguing perspectives. CD4^+^/CD8^+^, TSC, and Th.cell.Induced have been found to be associated with certain bacterial species, implying that bacteria have the potential to affect the adaptive immunity of patients. However, there are many intermediate issues to be dealt with before making a cohesive interpretation of this assumption. Combining the information from functional metagenomes and metabolomics will minimize the gap between gut microbiota and immunological responses of the recipients.

However, the findings of the study have to be seen in the light of some limitations. First, our samples that were collected within a certain region in China limit the generalization ability of our research findings. We could include samples from a greater geographic area or be expanded to a multicountry analysis. Second, the sample size of our cohort is relatively small, especially for UC cohort. Although some interesting findings have been found in our study, a larger sample size could allow us to compute confidence intervals besides *P*‐values in our statistical analysis and dig out more diversified species or strains which differs in abundance before and after FMT. Third, follow‐up time points of recipients after FMT in our study are within 1 month which is a short‐term scope. We could lengthen the follow‐ups to 6 months or 1 year to provide greater explanation regarding the stability of FMT in a mid‐term basis in the near future.

## Conclusions

The present attractive clinical findings are mainly based on our 1‐h FMT protocol for providing fresh FMT, which means the time from defecation of stool to deliver purified microbiota to patient’s intestine within 1 h 35, 36. Another factor contributing to this positive clinical response, according to our experience, might be the criteria of donor screening which is based on young age population, generally cover children and college students under 24‐years‐old 37.

Knowledge related to the mechanism of fecal microbiome transplantation in IBD patients is being accumulated as metagenomic and bioinformatics approaches to the microbiome and microbiome–phenotype association analysis. As our study revealed, microbiota alterations can be reflected at both species level and strain level after FMT. Strain‐level identification makes it possible to run toward the development of probiotics. In addition, the possibility of combining microbiota elements with phenotype factors to predict the gut microbiota composition of post‐FMT recipients has also been demonstrated. This highlights the value of utilizing advanced machine learning methods in investigating the principles of species engraftment.

## Conflict of interest

The authors declare no conflict of interest.

## Author contributions

ZJ, MZ, BC, and FZ conceived the study, contributed to methodology, and wrote the manuscript; FZ and HJ revised and edited the manuscript; BC, HW, and QF acquired the data; YZ, XZ, HY, and JW provided with material support; MZ and ZJ contributed software and formally analyzed the data; MZ and ZJ wrote the original draft of the manuscript; FZ and HJ supervised the study.

## Supporting information


**Fig. S1.** Three days after FMT, recipients who shared a donor gained varied amount of species count.

**Fig. S2.** Shift of species’ relative abundance across all recipients before and after FMT.

**Table S1.** Outcomes after FMT treatment.

**Table S2.** Spearman correlation coefficients and *P*‐value between clinical indexes’ change and diseases‐associated index.

## Data Availability

Datasets are in a publicly accessible repository: The quality‐controlled sequencing reads are available in the CNGB Nucleotide Sequence Archive (CNSA: https://db.cngb.org/cnsa; accession number CNP0000134).

## References

[1] Khor B , Gardet A and Xavier RJ (2011) Genetics and pathogenesis of inflammatory bowel disease. Nature 474, 307. 21677747 10.1038/nature10209 PMC3204665

[2] Manichanh C , Borruel N , Casellas F and Guarner F (2012) The gut microbiota in IBD. Nat Rev Gastroenterol Hepatol 9, 599. 22907164 10.1038/nrgastro.2012.152

[3] Halfvarson J , Brislawn CJ , Lamendella R , Vázquez‐Baeza Y , Walters WA , Bramer LM , D'Amato M , Bonfiglio F , McDonald D , Gonzalez A *et al* (2017) Dynamics of the human gut microbiome in inflammatory bowel disease. Nat Microbiol 2, 17004. 28191884 10.1038/nmicrobiol.2017.4 PMC5319707

[4] Sunkara T , Rawla P , Ofosu A and Gaduputi V (2018) Fecal microbiota transplant–a new frontier in inflammatory bowel disease. J Inflamm Res 11, 321. 30214266 10.2147/JIR.S176190 PMC6124474

[5] Sokol H , Pigneur B , Watterlot L , Lakhdari O , Bermúdez‐Humarán LG , Gratadoux JJ , Blugeon S , Bridonneau C , Furet JP , Corthier G *et al* (2008) *Faecalibacterium prausnitzii* is an anti‐inflammatory commensal bacterium identified by gut microbiota analysis of Crohn disease patients. Proc Natl Acad Sci USA 105, 16731–16736. 18936492 10.1073/pnas.0804812105 PMC2575488

[6] Mazmanian SK , Liu CH , Tzianabos AO and Kasper DL (2005) An immunomodulatory molecule of symbiotic bacteria directs maturation of the host immune system. Cell 122, 107–118. 16009137 10.1016/j.cell.2005.05.007

[7] Qiu X , Zhang M , Yang X , Hong N and Yu C (2013) *Faecalibacterium prausnitzii* upregulates regulatory T cells and anti‐inflammatory cytokines in treating TNBS‐induced colitis. J Crohns Colitis 7, e558–e568. 23643066 10.1016/j.crohns.2013.04.002

[8] Borody T , Torres M , Campbell J , Leis S and Nowak A (2011) Reversal of inflammatory bowel disease (IBD) with recurrent faecal microbiota transplants (FMT). Am J Gastroenterol 106, S366.

[9] Zhang FM , Wang HG , Wang M , Cui BT , Fan ZN and Ji GZ (2013) Fecal microbiota transplantation for severe enterocolonic fistulizing Crohn’s disease. World J Gastroenterol 19, 7213. 24222969 10.3748/wjg.v19.i41.7213 PMC3819561

[10] Gutin L , Piceno Y , Fadrosh D , Lynch K , Zydek M , Kassam Z , LaMere B , Terdiman J , Ma A , Somsouk M *et al* (2019). Fecal microbiota transplant for Crohn disease: a study evaluating safety, efficacy, and microbiome profile. United European Gastroenterol J 7, 807–814. 10.1177/2050640619845986 PMC6620877 31316785

[11] Paramsothy S , Kamm MA , Kaakoush NO , Walsh AJ , van den Bogaerde J , Samuel D , Leong RWL , Connor S , Ng W , Paramsothy R *et al* (2017) Multidonor intensive faecal microbiota transplantation for active ulcerative colitis: a randomised placebo‐controlled trial. Lancet 389, 1218–1228. 28214091 10.1016/S0140-6736(17)30182-4

[12] Moayyedi P , Surette MG , Kim PT , Libertucci J , Wolfe M , Onischi C , Armstrong D , Marshall JK , Kassam Z , Reinisch W *et al* (2015) Fecal microbiota transplantation induces remission in patients with active ulcerative colitis in a randomized controlled trial. Gastroenterology 149, 102–109. 25857665 10.1053/j.gastro.2015.04.001

[13] Rossen NG , Fuentes S , van der Spek MJ , Tijssen JG , Hartman JH , Duflou A , Löwenberg M , van den Brink GR , Mathus‐Vliegen EM , de Vos WM *et al* (2015) Findings from a randomized controlled trial of fecal transplantation for patients with ulcerative colitis. Gastroenterology 149, 110–118. 25836986 10.1053/j.gastro.2015.03.045

[14] Colman RJ and Rubin DT (2014) Fecal microbiota transplantation as therapy for inflammatory bowel disease: a systematic review and meta‐analysis. J Crohns Colitis 8, 1569–1581. 25223604 10.1016/j.crohns.2014.08.006 PMC4296742

[15] Angelberger S , Reinisch W , Makristathis A , Lichtenberger C , Dejaco C , Papay P , Novacek G , Trauner M , Loy A and Berry D (2013) Temporal bacterial community dynamics vary among ulcerative colitis patients after fecal microbiota transplantation. Am J Gastroenterol 108, 1620. 24060759 10.1038/ajg.2013.257

[16] Gordon H and Harbord M (2014) A patient with severe Crohn's colitis responds to faecal Microbiota transplantation. J Crohns Colitis 8, 256–257. 24239403 10.1016/j.crohns.2013.10.007

[17] Cui B , Feng Q , Wang H , Wang M , Peng Z , Li P , Huang G , Liu Z , Wu P , Fan Z *et al* (2015) Fecal microbiota transplantation through mid‐gut for refractory Crohn's disease: safety, feasibility, and efficacy trial results. J Gastroenterol Hepatol 30, 51–58. 25168749 10.1111/jgh.12727

[18] Suskind DL , Brittnacher MJ , Wahbeh G , Shaffer ML , Hayden HS , Qin X , Singh N , Damman CJ , Hager KR , Nielson H *et al* (2015) Fecal microbial transplant effect on clinical outcomes and fecal microbiome in active Crohn's disease. Inflamm Bowel Dis 21, 556–563. 25647155 10.1097/MIB.0000000000000307 PMC4329080

[19] Frank DN , Amand ALS , Feldman RA , Boedeker EC , Harpaz N and Pace NR (2007) Molecular‐phylogenetic characterization of microbial community imbalances in human inflammatory bowel diseases. Proc Natl Acad Sci USA 104, 13780–13785. 17699621 10.1073/pnas.0706625104 PMC1959459

[20] Hansen R , Russell RK , Reiff C , Louis P , McIntosh F , Berry SH , Mukhopadhya I , Bisset WM , Barclay AR , Bishop J *et al* (2012) Microbiota of de‐novo pediatric IBD: increased *Faecalibacterium prausnitzii* and reduced bacterial diversity in Crohn's but not in ulcerative colitis. Am J Gastroenterol 107, 1913. 23044767 10.1038/ajg.2012.335

[21] Bron PA , Van Baarlen P and Kleerebezem M (2012) Emerging molecular insights into the interaction between probiotics and the host intestinal mucosa. Nat Rev Microbiol 10, 66. 10.1038/nrmicro2690 22101918

[22] Needham BD , Carroll SM , Giles DK , Georgiou G , Whiteley M and Trent MS (2013) Modulating the innate immune response by combinatorial engineering of endotoxin. Proc Natl Acad Sci USA 110, 1464–1469. 23297218 10.1073/pnas.1218080110 PMC3557076

[23] Spanogiannopoulos P , Bess EN , Carmody RN and Turnbaugh PJ (2016) The microbial pharmacists within us: a metagenomic view of xenobiotic metabolism. Nat Rev Microbiol 14, 273. 26972811 10.1038/nrmicro.2016.17 PMC5243131

[24] Smillie CS , Sauk J , Gevers D , Friedman J , Sung J , Youngster I , Hohmann EL , Staley C , Khoruts A , Sadowsky MJ *et al* (2018) Strain tracking reveals the determinants of bacterial engraftment in the human gut following fecal microbiota transplantation. Cell Host Microbe 23, 229–240. 29447696 10.1016/j.chom.2018.01.003 PMC8318347

[25] Vrieze A , Van Nood E , Holleman F , Salojärvi J , Kootte RS , Bartelsman JF , Dallinga‐Thie GM , Ackermans MT , Serlie MJ , Oozeer R *et al* (2012) Transfer of intestinal microbiota from lean donors increases insulin sensitivity in individuals with metabolic syndrome. Gastroenterology 143, 913–916. 22728514 10.1053/j.gastro.2012.06.031

[26] Fang C , Zhong H , Lin Y , Chen B , Han M , Ren H , Lu H , Luber JM , Xia M , Li W *et al* (2017). Assessment of the cPAS‐based BGISEQ‐500 platform for metagenomic sequencing. Gigascience 7, gix133. 10.1093/gigascience/gix133 PMC5848809 29293960

[27] Sunagawa S , Mende DR , Zeller G , Izquierdo‐Carrasco F , Berger SA , Kultima JR , Coelho LP , Arumugam M , Tap J , Nielsen HB *et al* (2013) Metagenomic species profiling using universal phylogenetic marker genes. Nat Methods 10, 1196. 24141494 10.1038/nmeth.2693

[28] Costea PI , Munch R , Coelho LP , Paoli L , Sunagawa S and Bork P (2017) metaSNV: a tool for metagenomic strain level analysis. PLoS ONE 12, e0182392. 28753663 10.1371/journal.pone.0182392 PMC5533426

[29] Liaw A and Wiener M (2002) Classification and regression by randomForest. R News 2, 18–22.

[30] Shannon P , Markiel A , Ozier O , Baliga NS , Wang JT , Ramage D , Amin N , Schwikowski B and Ideker T (2003) Cytoscape: a software environment for integrated models of biomolecular interaction networks. Genome Res 13, 2498–2504. 14597658 10.1101/gr.1239303 PMC403769

[31] Zhou Y and Zhi F (2016). Lower level of bacteroides in the gut microbiota is associated with inflammatory bowel disease: a meta‐analysis. BioMed Res Int, 2016, 5828959. 10.1155/2016/5828959 PMC5143693 27999802

[32] Li SS , Zhu A , Benes V , Costea PI , Hercog R , Hildebrand F , Huerta‐Cepas J , Nieuwdorp M , Salojärvi J , Voigt AY *et al* (2016) Durable coexistence of donor and recipient strains after fecal microbiota transplantation. Science 352, 586–589. 27126044 10.1126/science.aad8852

[33] Neville BA and O’Toole PW (2010) Probiotic properties of *Lactobacillus salivarius* and closely related Lactobacillus species. Future Microbiol 5, 759–774. 20441548 10.2217/fmb.10.35

[34] Deng H , Li Z , Tan Y , Guo Z , Liu Y , Wang Y , Yuan Y , Yang R , Bi Y , Bai Y *et al* (2016) A novel strain of *Bacteroides fragilis* enhances phagocytosis and polarises M1 macrophages. Sci Rep 6, 29401. 27381366 10.1038/srep29401 PMC4933912

[35] Wang H , Cui B , Li Q , Ding X , Li P , Zhang T , Yang X , Ji G and Zhang F (2018) The safety of fecal microbiota transplantation for Crohn’s disease: findings from a long‐term study. Adv Ther 35, 1935–1944. 30328062 10.1007/s12325-018-0800-3 PMC6223988

[36] Ding X , Li Q , Li P , Zhang T , Cui B , Ji G , Lu X and Zhang F (2019) Long‐term safety and efficacy of fecal microbiota transplant in active ulcerative colitis. Drug Saf 42, 869–880. 30972640 10.1007/s40264-019-00809-2

[37] Zhang F , Zhang T , Zhu H and Borody TJ (2019) Evolution of fecal microbiota transplantation in methodology and ethical issues. Curr Opin Pharmacol 49, 11–16. 31059962 10.1016/j.coph.2019.04.004

