# Supplementary figures and images for: Fecal microbiota transplantation results in bacterial strain displacement in patients with inflammatory bowel diseases
Source: FEBS Open Bio. 2019 Dec 13;10(1):41–55. doi: 10.1002/2211-5463.12744 (PMC6943227; doi:10.1002/2211-5463.12744)

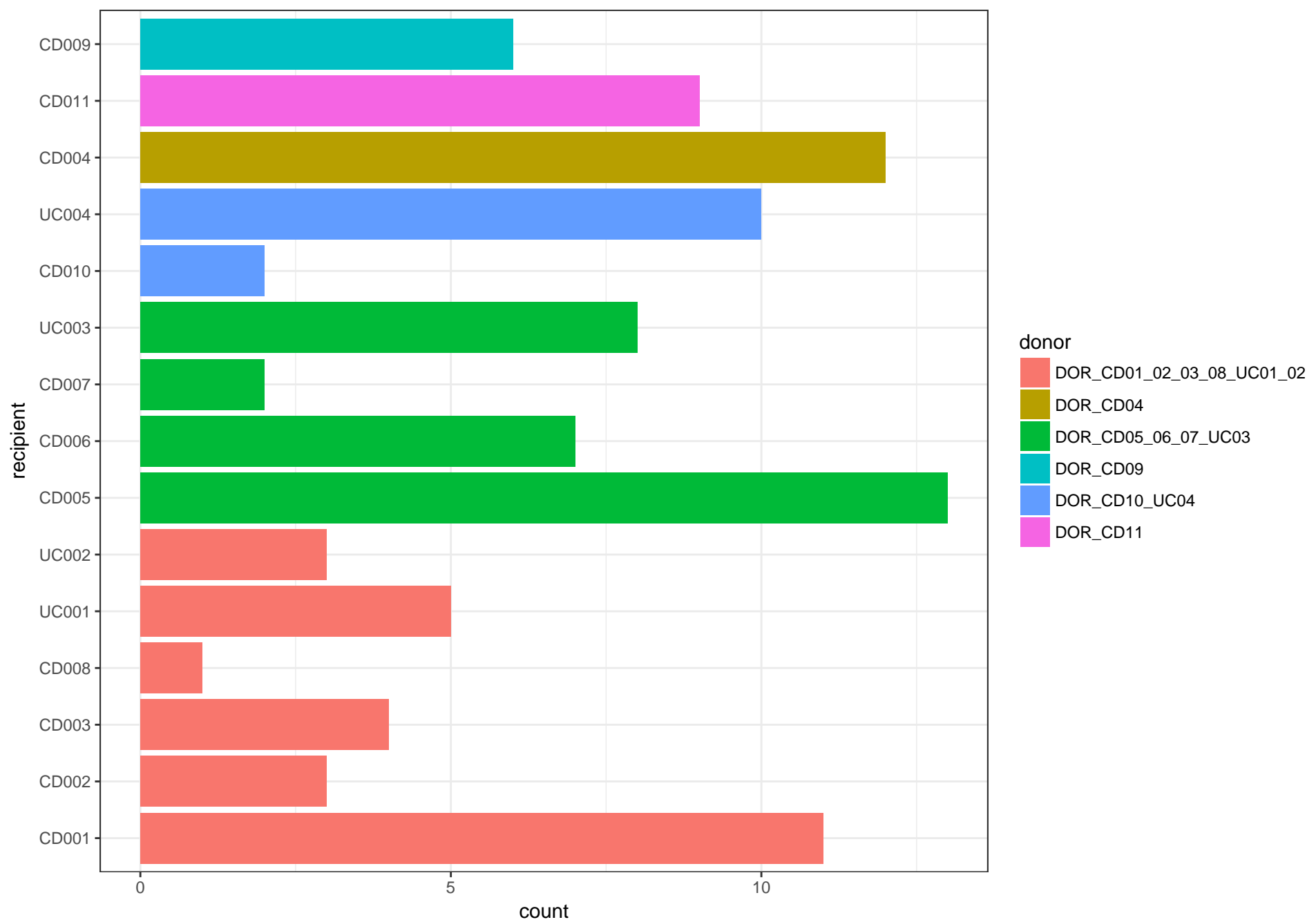

Supplement: Supplementary file 1 — Fig. S1. Three days after FMT, recipients who shared a donor gained varied amount of species count. [file FEB4-10-41-s001.pdf]

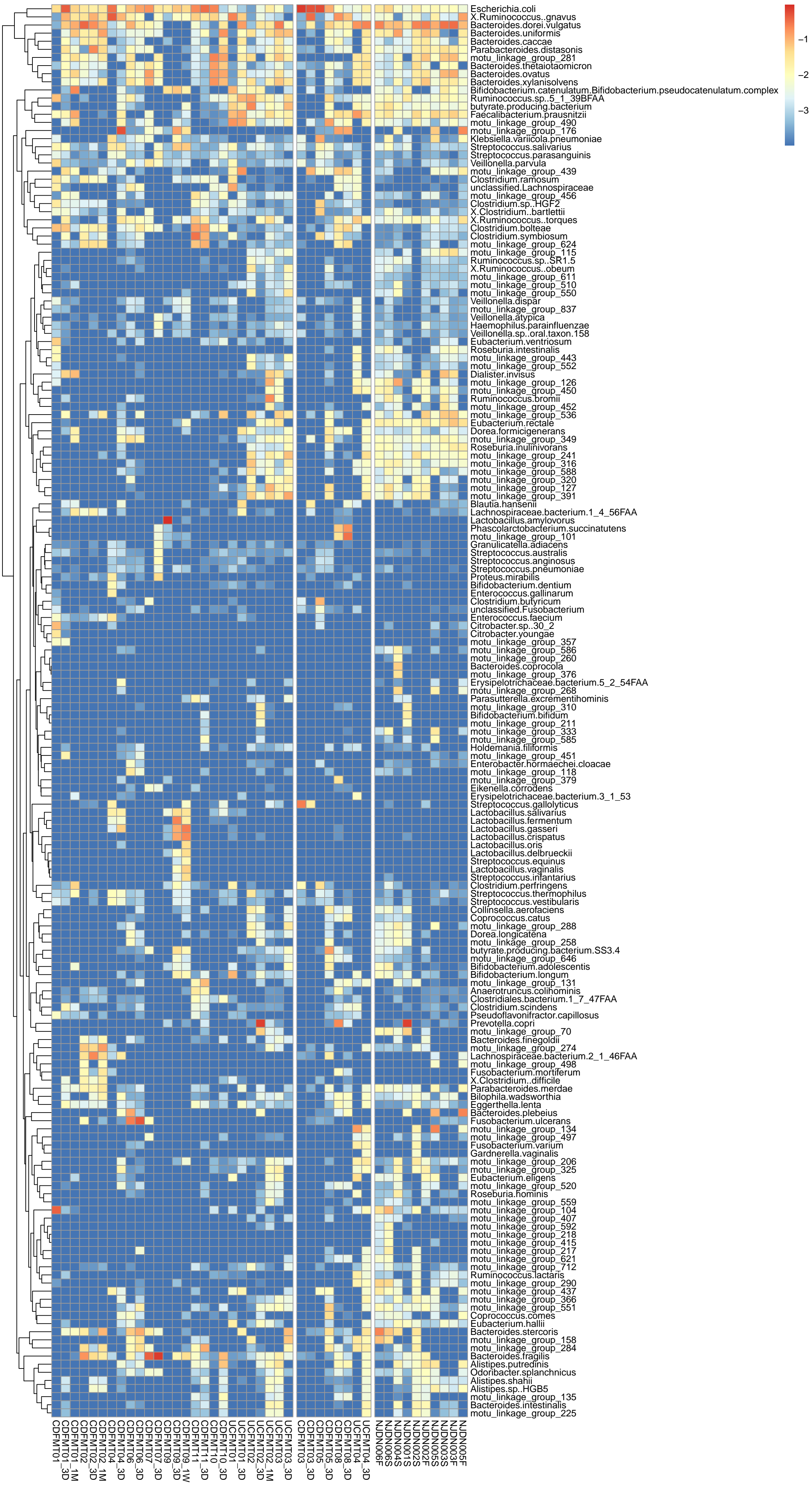

Supplement: Supplementary file 2 — Fig. S2. Shift of species’ relative abundance across all recipients before and after FMT. [file FEB4-10-41-s002.pdf]
