# Supplementary material for: Fecal microbiota transplantation results in bacterial strain displacement in patients with inflammatory bowel diseases
Source: FEBS Open Bio. 2019 Dec 13;10(1):41–55. doi: 10.1002/2211-5463.12744 (PMC6943227; doi:10.1002/2211-5463.12744)
Supplement: Supplementary file 3 — Table S1. Outcomes after FMT treatment. [file FEB4-10-41-s003.docx]

Table S1 Outcomes after FMT treatment

| Patient | Donor | Relieved | Healed | Fever |
| --- | --- | --- | --- | --- |
| CD001FMT3D | NJDN003S | Yes | --- | No |
| CD001FMT1M | NJDN003S | Yes | Yes | No |
| CD002FMT3D | NJDN003S | Yes | --- | No |
| CD002FMT1M | NJDN003S | Yes | --- | No |
| CD003FMT3D | NJDN003S | No | --- | Releaved |
| CD004FMT3D | NJDN005S | Yes | --- | Releaved |
| CD005FMT3D | NJDN006S | No | --- | No |
| CD006FMT3D | NJDN006S | Yes | Yes | No |
| CD007FMT3D | NJDN006S | Yes | No | No |
| CD008FMT3D | NJDN003S | No | No | Releaved |
| CD009FMT3D | NJDN004S | Yes | --- | No |
| CD009FMT1W | NJDN004S | Yes | --- | No |
| CD010FMT3D | NJDN002S | Yes | Yes | No |
| CD011FMT3D | NJDN001S | Yes | --- | No |
| UC001FMT3D | NJDN003S | Yes | No | No |
| UC002FMT3D | NJDN003S | Yes | Yes | No |
| UC002FMT1M | NJDN003S | Yes | Yes | No |
| UC003FMT3D | NJDN006S | Yes | Yes | No |
| UC004FMT3D | NJDN002S | No | No | No |

Note. “---” means missing data
