# Supplementary material for: Fecal microbiota transplantation results in bacterial strain displacement in patients with inflammatory bowel diseases
Source: FEBS Open Bio. 2019 Dec 13;10(1):41–55. doi: 10.1002/2211-5463.12744 (PMC6943227; doi:10.1002/2211-5463.12744)
Supplement: Supplementary file 4 — Table S2. Spearman correlation coefficients and P‐value between clinical indexes’ change and diseases‐associated index. [file FEB4-10-41-s004.docx]

Table S2 Spearman correlation coefficients and P-value between clinical indexes’ change and diseases-associated indexes

|  | age | weight | durance | Start age | BMI | recip_shann | donor_shann | shann_mean |
| --- | --- | --- | --- | --- | --- | --- | --- | --- |
| stool | 0.28 | 0.18 | 0.94 | 0.32 | 0.34 | 0.78 | 0.11 | 0.24 |
| Abdominal_pain_score_change | 0.08 | 0.23 | 0.42 | 0.04 | 0.06 | 0.29 | 0.21 | 0.87 |
| Defecation_change | 0.18 | 0.32 | 0.28 | 0.24 | 0.32 | 0.57 | 0.40 | 0.32 |
| Mucous_stool_change | 0.93 | 0.79 | 0.66 | 0.94 | 0.51 | 0.79 | 0.82 | 0.71 |
| Bloody_purulent_stool_change | 0.28 | 0.18 | 0.94 | 0.32 | 0.34 | 0.78 | 0.11 | 0.24 |
| Stool_form_change | 0.56 | 0.84 | 0.36 | 0.45 | 0.67 | 0.88 | 0.17 | 0.35 |
| Tenesmus_change | 0.67 | 0.38 | 0.32 | 0.82 | 0.58 | 0.69 | 0.13 | 0.22 |
| Appetite_change | 0.21 | 0.98 | 0.38 | 0.50 | 0.61 | 0.14 | 0.94 | 0.23 |
| Mental_status_change | 0.84 | 0.55 | 0.56 | 0.81 | 0.47 | 0.10 | 0.91 | 0.14 |
| T.cell_change | 0.08 | 0.27 | 0.37 | 0.32 | 0.13 | 0.96 | 0.99 | 0.97 |
| B.cell_change | 0.71 | 0.74 | 0.32 | 0.15 | 0.71 | 0.71 | 0.36 | 0.82 |
| Th.cell.Induced_change | 0.00 | 0.20 | 0.47 | 0.02 | 0.08 | 0.31 | 0.89 | 0.44 |
| TSC_change | 0.18 | 0.93 | 0.87 | 0.05 | 0.58 | 0.08 | 0.56 | 0.27 |
| NK.cell_change | 0.09 | 0.65 | 0.79 | 0.10 | 0.22 | 0.95 | 0.68 | 0.85 |
| CD4._CD8._change | 0.01 | 0.58 | 0.78 | 0.01 | 0.18 | 0.11 | 0.41 | 0.41 |

|  | age | weight | durance | Start age | BMI | recip_shann | donor_shann | shann_mean |
| --- | --- | --- | --- | --- | --- | --- | --- | --- |
| Stool | -0.31 | -0.25 | 0.02 | -0.12 | -0.24 | 0.14 | 0.46 | 0.28 |
| Abdominal_pain_score_change | -0.47 | -0.26 | 0.17 | -0.49 | -0.47 | -0.29 | 0.21 | -0.11 |
| Defecation_change | 0.35 | 0.19 | 0.15 | 0.17 | 0.10 | -0.19 | -0.26 | -0.32 |
| Mucous_stool_change | -0.06 | 0.03 | -0.09 | -0.15 | -0.21 | -0.16 | -0.14 | -0.15 |
| Bloody_purulent_stool_change | -0.31 | -0.25 | 0.02 | -0.12 | -0.24 | 0.14 | 0.46 | 0.28 |
| Stool_form_change | -0.05 | -0.03 | -0.05 | -0.17 | -0.13 | -0.13 | -0.35 | -0.22 |
| Tenesmus_change | 0.15 | 0.24 | 0.11 | -0.01 | 0.07 | -0.28 | -0.39 | -0.39 |
| Appetite_change | 0.32 | -0.14 | 0.40 | 0.12 | 0.06 | -0.41 | 0.07 | -0.30 |
| Mental_status_change | 0.17 | -0.22 | 0.07 | 0.16 | -0.17 | -0.51 | 0.11 | -0.35 |
| T.cell_change | -0.24 | -0.14 | -0.34 | 0.07 | -0.31 | -0.23 | 0.12 | -0.24 |
| B.cell_change | 0.10 | 0.07 | -0.07 | -0.25 | 0.07 | 0.15 | -0.24 | 0.01 |
| Th.cell.Induced_change | -0.61 | -0.42 | -0.29 | -0.37 | -0.36 | 0.28 | 0.21 | 0.39 |
| TSC_change | 0.29 | 0.16 | 0.10 | 0.47 | 0.08 | -0.52 | 0.09 | -0.53 |
| NK.cell_change | 0.06 | -0.23 | 0.37 | 0.02 | -0.17 | 0.00 | 0.07 | 0.15 |
| CD4._CD8._change | -0.36 | -0.09 | -0.22 | -0.40 | -0.07 | 0.50 | -0.24 | 0.39 |

Note. reip_shann = Shannon index of FMT recipient; donor_shann = Shannon index of FMT donor;

shann_mean = the average Shannon index of recipient and donor’ Shannon index;

durance = disease during; Start age = disease start age;

p < 0.05 was considered as significant
