# Supplementary material for: Fecal microbiota transplantation results in bacterial strain displacement in patients with inflammatory bowel diseases
Source: FEBS Open Bio. 2019 Dec 13;10(1):41–55. doi: 10.1002/2211-5463.12744 (PMC6943227; doi:10.1002/2211-5463.12744)
Supplement: Supplementary file 5 [file FEB4-10-41-s005.docx]

Fig. S1. The amount of donor-specific species gain after FMT differs, even for same-donor recipients. Recipients that share a donor are colored the same.

Fig. S2. A certain number of donor species display apparent transfer after FMT treatment in IBD patients. Heatmap and hierarchical clustering of mOTU profiles for all samples. Pre- and post-FMT CD recipients, pre- and post-FMT UC recipients, and healthy controls are separated by space.
